# Phenolic Profile, Antioxidant Activity, and Ameliorating Efficacy of *Chenopodium quinoa* Sprouts against CCl_4_-Induced Oxidative Stress in Rats

**DOI:** 10.3390/nu12102904

**Published:** 2020-09-23

**Authors:** Maryam M. Al-Qabba, Maha A. El-Mowafy, Sami A. Althwab, Hani A. Alfheeaid, Thamer Aljutaily, Hassan Barakat

**Affiliations:** 1 Department of Food Science and Human Nutrition, College of Agriculture and Veterinary Medicine, Qassim University, Buraydah 51452, Saudi Arabia; m.m.g.2141@gmail.com (M.M.A.-Q.); dr.maha.2009@gmail.com (M.A.E.-M.); thaoab@qu.edu.sa (S.A.A.); h.alfheeaid@qu.edu.sa (H.A.A.); thamer.aljutaily@qu.edu.sa (T.A.); 2 School of Medicine, Dentistry and Nursing, College of Medical, Veterinary and Life Sciences, University of Glasgow, Glasgow G12 8QQ, UK; 3 Food Technology Department, Faculty of Agriculture, Benha University, Moshtohor 13736, Egypt

**Keywords:** *Chenopodium quinoa*, sprouts, polyphenols, oxidative stress, antioxidant activity, amelioration efficacy

## Abstract

Quinoa (*Chenopodium quinoa*) is classified as one of the pseudo-cereal grains rich in both macronutrients and micronutrients. This study tracks changes in the polyphenol composition of red quinoa (RQ) and yellow quinoa (YQ) seeds during germination. The antioxidant bioactivity of raw and germinated seed was also determined in vitro. Phenolic acids and their derivatives and flavonoids were identified by using HPLC-DAD and quantified after 0, 3, and 6 days of germination. Subsequently, the extracts of 6-day-old quinoa sprouts were prepared to biologically evaluate their functional properties against CCl_4_-induced oxidative stress in rats. The results indicated that antioxidant activity (AOA) of total phenolic compounds (TPC), and flavonoids significantly increased in RQ and YQ sprouts during germination up to 9 days. RQ sprouts exhibited stronger bioactive compound diversity than YQ sprouts as observed in HPLC analysis. Among the 11 and 8 quantified polyphenols, ferulic acid and quercetin were predominant phenolic acid and flavonoid in RQ and YQ sprouts, respectively. After 6 days of germination, 16 and 8 polyphenols were detected and quantified in RQ and YQ sprouts, respectively. Interestingly, the treatment of rats at a dose of 30 mg of Gallic acid Equivalent (GAE) kg^−1^ significantly reduced fasting blood glucose (FBG), alanine aminotransferase (ALT), aspartate aminotransferase AST, and total bilirubin (TIBIL) and improved liver inflammation. Furthermore, RQ and YQ sprouts improved the blood profile by significantly decreasing low-density lipoproteins (LDL) and very low-density lipoproteins (VLDL) and increasing high-density lipoproteins (HDL). Moreover, RQ and YQ sprout extracts significantly reduced malonaldehyde (MDA) and efficiently enhanced the reduced glutathione (GSH) and superoxide dismutase (SOD) activities in oxidative stress-induced rats as compared to CCl_4_-rats. In conclusion, red quinoa sprouts (RQS) and yellow quinoa sprouts (YQS) provide naturally synthesized polyphenols, possessing superior antioxidant activity, and their ethanolic extracts have promising effects and potential health benefits to counter induced oxidative stress. Incorporating quinoa sprouts as functional food ingredients should be considered and scaling-up its production is beneficial.

## 1. Introduction

Oxidative stress refers to an imbalance between the occurrence of reactive oxygen species and detoxification or repairing mechanisms inside the body [1]. Disturbance in the normal redox state of cells produces toxic peroxides and free radicals that damage cell lipids, proteins, and DNA [2]. Oxidative stress from oxidative metabolism can break DNA strands to cause underlying damage. The indirect base damage of reactive oxygen species (ROS) occurs by generating hydroxyl radical, superoxide radical, and hydrogen peroxide [2]. The oxidative stress can disrupt normal cellular signaling as some reactive oxidative species also act as cellular messengers in redox signaling. In humans, oxidative stress is considered a potential cause of cancer, Alzheimer’s disease [1], atherosclerosis [3], and depression [4]. Researchers are focusing on finding healthy and efficient plant-based bioactive suppressive compounds against oxidative stress complications.

Quinoa seeds (*Chenopodium quinoa* Willd.) have received growing attention in recent years. Quinoa seeds contain natural antioxidant elements and phytochemicals that are vital for human health [5,6]. The nutritional value and functional properties of quinoa (especially gluten-free) may benefit children, adults, the elderly, and women exposed to osteoporosis and people with anemia, diabetes, high blood fat, obesity, or celiac disease [7]. Vitamin content and polyphenols were significantly increased after 72 h of germination in comparison to raw quinoa seeds, whereas fermentation decreased both compounds. Phenolic compounds and antioxidant capacity were noticeably improved during the germination process [8,9]. The germination process naturally produced enriched ingredients with health-promoting antioxidant compounds [8,10,11]. In a study, quinoa seeds reduced oxidative stress in the plasma, heart, lung, liver, kidney, testicle, spleen, and pancreas of mice fed with fructose. Quinoa seeds also inhibited fat peroxidation, lowered plasma malonaldehyde (MDA) concentrations, and maintained the normal activity of superoxide dismutase (SOD), catalase (CAT), and glutathione peroxidase (GPx) enzymes [8,12].

The ameliorating efficacy of *C. quinoa* sprouts against CCl_4_-induced oxidative stress in rats has not yet been studied. Therefore, the total phenolic compounds (TPC), antioxidant activity (AOA), total carotenoids (TC), total saponins (TS), total anthocyanins (TA), total flavonoids (TF), and total flavonols (TFL) were studied, and HPLC analysis of phenolic compounds during germination was carried out. Furthermore, the protective effects of ethanolic extracts of both red quinoa sprouts (RQS) and yellow quinoa sprouts (YQS) after 6 days of germination against CCl_4_-induced oxidative stress in rats were investigated.

## 2. Materials and Methods

### 2.1. Animals and Raw Quinoa Seeds

Wistar rats (30 adult males) weighing between 150–175 g were used in this investigation. All experiments were approved by the Institutional Animal Ethics Committee (IAEC) of Qassim University, KSA, which were regulated by the Control and Supervision of Experiments on Animals (CPCSEA) Committee under the National Committee of Bio-Ethics (NCBE) that implements regulations related to the ethics of research on living creatures. Animals were housed in air-conditioned polypropylene cages and kept under standard laboratory conditions at 25 ± 1 °C.

RQ and YQ seeds (*Chenopodium quinoa* Will.) were purchased from the Al-Tamimi market at Al Qassim region, KSA. The YQ seeds were cultivated in Peru and exported by Bode Naturkost Co., whereas the RQ seeds were cultivated in the USA and exported by living Now Inc. Co. Dust and broken and diseased seeds were removed. Cleaned seeds were immediately grown to produce quinoa sprouts. Raw or milled seeds (Snijders Scientific Tilburg; Model: 8010E, Holland) were kept in freezer-plastic bags and stored at −18 ± 1 °C until used for analysis.

### 2.2. Germination Process of Quinoa Seeds

Germination was performed in the batches of 100 g seeds, which were sanitized by soaking in a 2% sodium hypochlorite solution for 2 min. Seeds were washed 3 times with sterilized distilled water (sd.H_2_O) and uniformly spread between a double layer of wet filter paper in 10 cm Petri dishes (10 g quinoa seeds in each). Twelve Petri dishes containing quinoa seeds were placed into a plastic box (32 × 22 × 8 cm) and loaded in the seed germinator. The germination process was carried out inside a temperature-controlled seed germinator equipped with an atomizer (EasyGreen, Model: EGL 50, Canada) at 17 ± 1 °C and 90% relative humidity under dark conditions. Quinoa samples were daily sprayed with 0.3 mL sd.H_2_O/dish for the first 3 days. Samples were collected after 3, 6, and 9 days from the beginning of the germination process. Quinoa sprouts were immediately frozen overnight at –18 ± 1 °C, freeze-dried for 96 h at −48 °C (CHRIST, Alpha 1–2 LD plus, Germany), and 0.032 mbar. Freeze-dried sprouts were ground in a small mill (Snijders Scientific Tilburg; Model: 8010E, Holland), sieved (60 mesh sieve) to prepare homogeneous powder that was kept in dark packages at 4 ± 1 °C until phytochemicals and HPLC analyses. To conduct the biological assessment of quinoa sprouts, 2 kg of each RQ and YQ was separately germinated under the same conditions for 6 days, gradually dried by following a 24-h drying program according to Barakat et al. [13], milled, sieved, and kept under cold storage until extraction.

### 2.3. Quinoa Seeds Extraction

Two types of quinoa sprouts powder were extracted with 70% ethanol (1:5 *w*:*v*) at 120 rpm for 2 h at room temperature (25 °C) and the resulting residue was re-extracted twice under the same conditions. The ethanolic extracts were evaporated under vacuum (BÜCHI Rotary Evaporators R210, Germany) at controlled temperature (40 °C) until complete removal of the solvent. The TPC content of RQS and YQS ethanolic extracts was determined according to Bettaieb et al. [14] and then the extracts were frozen until used for rat’s administration. A volume containing 30 mg GAE kg^−1^ of rat body weight was quantified according to Reagan-Shaw et al. [15] for oral administration.

### 2.4. Determination of TPC, TC, TS, TA, TF, and TFL during Germination

The TPC of raw seeds and sprouts of *C. quinoa* was determined by using Folin–Ciocalteu reagent according to Yawadio Nsimba et al. [15]. The measurements were compared to the standard curve of Gallic acid (GA) solution (*R*^2^ = 0.99), and TPC content was expressed as milligrams of Gallic acid equivalents (GAE) per 100 g (mg of GAE 100 g^−1^ DW). To determine the TC, 5 g quinoa or freeze-dried sprouts were repeatedly extracted with a mixture of acetone and petroleum ether (1:1, *v*/*v*) according to Yuan et al. [16]. The upper phase was collected, washed several times with water, and combined with crude extracts. Petroleum ether was added in the solution to prepare a known volume. TC content was spectrophotometrically determined at 451 nm as mg 100 g^−1^ DW. The TS was quantified using the standard afrosimetric method according to Koziol [17], and the results were expressed as mg g^−1^ quinoa on dry base weight (mg g^−1^ DW). The TA content was determined by the pH differential method following the protocol of Giusti and Wrolstad [18] and expressed as mg 100 g^−1^ DW. The TF content of quinoa or freeze-dried sprouts was determined according to Mohdaly et al. [19] at 440 nm and expressed as mg quercetin equivalent (QE) per 100 g^−1^ (mg QE 100 g^−1^ DW).

### 2.5. Free Radical Scavenging Ability on DPPH

Radical scavenging activity was measured spectrophotometrically, based on the bleaching of DPPH radicals purple solution according to Yawadio Nsimba et al. [20]. The percentage of DPPH radical scavenging activity was used to plot the Trolox calibration curve. The antiradical activity was expressed as micromoles of Trolox Equivalents (TE) per gram (µmol TE g^−1^).

### 2.6. Identification of Phenolic Compounds in Quinoa Sprouts during Germination by HPLC-DAD

During the 6 days of germination, the phenolic compounds of RQ, YQ, and their sprouts were determined by HPLC system HP1100 (Agilent Technologies, Palo Alto, CA, USA) equipped with an auto-sampler, quaternary pump, and diode array detector DAD (Hewlett Packard 1050), using a column (Altima C18, 5 × 150 mm, 4.6 mm id) and a guard column (Altima C18, 5 mm) according to Kim et al. [21]. The solvent system contained a gradient of A (Acetic acid 2.5%), B (Acetic acid 8%), and C (acetonitrile). The 10 µL of solvent was injected at a flow rate of 1 mL min^−1^ and separation was performed at 25 °C. The peaks of phenolic compounds (µg g^−1^ DW) were identified by comparing the results with the retention times and spectra of library and external standards. The external standards were purchased from Sigma-Aldrich, St Louis, MO, USA.

### 2.7. Biological Assessment of RQS and YQS Extracts In Vivo

After 10 d of acclimatization, rats were randomly divided into 5 groups (6 rats/group), housed in new cages under controlled conditions at 25 ± 2 °C, 40–45% relative humidity, and a 12-h light/dark cycle. The rats were labeled, and body weight was recorded. The rats were fed on a commercial standard pellet diet and water ad libitum. Different groups of rats were treated as follows: Group I (NR)—received an intraperitoneal injection of fresh olive oil (1.0 mL kg^−1^) twice per week and 0.5 mL distilled water orally per day; Group II (CCl_4_-R)—received an intraperitoneal injection of a fresh mixture of equal volumes of CCl_4_ and olive oil (1.0 mL kg^−1^) twice per week according to Chen et al. [9] with a minor modification; Group III (CCl_4_-R + Si-Vit E)—received an intramuscular injection of vitamin E and Selenium (E & Se) (Selepherol, Vetoquinol Co., Magny-Vernois, France) at a dose of 30 mg kg^−1^ twice per week, CCl_4_ twice per week and 1 mL distilled water orally per day; Group IV (CCl_4_-R + YQS)—received 30 mg GAE kg^−1^ YQS extract orally administrated daily for 4 weeks along with CCl_4_ twice per week; Group V (CCl_4_-R + RQS)—received 30 mg GAE kg^−1^ RQS extract orally administrated daily for 4 weeks along with CCl_4_ twice per week. The 12 h-starved rats were anesthetized with a mixture of alcohol: chloroform: ether (1:2:3). Blood samples were collected from the heart puncture of all the animals and each blood sample was subdivided into two tubes. Half tubes were subjected to serum separation by centrifugation and kept at –18 ± 1 °C for screening oxidative stress indices and biochemical parameters. The relative weight of the liver and kidney was calculated according to Zafar and Naqvi [22] at the end of the experimental period. Briefly, the rats were dissected to collect livers, and kidneys, which were twice washed with saline solution and dried using tissue papers. Livers and kidneys were weighed to determine the weight change of the organs relative to their body weights.

### 2.8. Determination of Fasting Glucose Blood Level, Liver Functions, and Lipid Profile

Fasting blood glucose (FBG) level was determined using a Glucose meter (USA). Alanine aminotransferase (ALT), total bilirubin (TBILI), and aspartate aminotransferase (AST) in blood serum were examined in Hitachi 902 Auto-analyzer (Boehringer Mannheim, Mannheim, Germany). Lipid profile, including triglycerides (TG), total cholesterol (TCh), and high-density lipoproteins (HDL) were also determined in the Hitachi 902 Auto-analyzer (Boehringer Mannheim, Mannheim, Germany). Low-density lipoproteins (LDL) were calculated according to Wilson (1998) whereas very-low-density lipoproteins (VLDL) were calculated according to Friedewald et al. [23].

### 2.9. Oxidative Stress Markers

Lipid peroxidation was estimated by measuring TBARS and expressed in terms of MDA content, according to Ohkawa et al. [24]. MDA, an end product of fatty acid peroxidation, forms a colored complex by reacting with thiobarbituric acid (TBA). The absorbance of the supernatant was measured at 532 nm and the results were calculated as nmol MDA mL^−1^. SOD activity was determined according to Giannopolitis and Ries [25] based on the ability of SOD to inhibit the autoxidation of adrenaline to adrenochrome. The color reaction was measured at 480 nm and expressed as U L^−1^. Reduced glutathione (GSH) was estimated according to the method described by Beutler et al. [26].

### 2.10. Statistical Analysis

Statistical analysis was performed using SPSS 22.0 for Windows. Experimental results were expressed as mean ± SE. Statistical significance was tested with one-way ANOVA followed by post hoc test and *p*-values < 0.05 were applied according to Steel et al. [27].

## 3. Results

The results presented in Table 1 summarize the TPC, AOA, TC, TS, TA, TF, and TFL contents in raw seeds and sprouts (grown at 17 ± 1 °C and 90–93% RH) during 9 days of germination. The TPC was significantly (*p* < 0.05) higher in raw seeds of YQ than RQ but significantly increased only in RQ during germination (*p* < 0.05) as compared to YQ. TPC increasing rate reached up to 144.40%, 178.90%, and 138.9% and 69.39%, 130.40%, and 97.24% in RQ and YQ after 3, 6, and 9 days, respectively. The AOA of RQ was significantly higher than YQ in raw seeds and sprouts and the highest value was recorded on the sixth day of germination (Table 1). The TC content gradually increased in both quinoa types during germination and was noted to be higher in raw YQ than raw RQ. The TS content did not significantly differ between raw RQ and YQ seeds. Interestingly, the germination process gradually decreased the TS content at a rate of 6.6%, 59.3%, and 85.8% in RQ and 42.3%, 84.8%, and 91.8% in YQ after 3, 6, and 9 days, respectively. The TA content in raw RQ seeds was noted as 179.96 mg 100 g^−1^, which drastically decreased by 10.6%, 30%, and 48.9% after 3, 6, and 9 days, respectively (Table 1). The initial TF content was increased by 36%, 47.2%, and 86.8% in RQ and 112.8%, 165.4%, and 169.4% in YQ after 3, 6, and 9 days, respectively. Additionally, the TFL content was increased with the germination period by 22.9%, 37.0%, and 59.1% in RQ and 199.7%, 459.8%, and 502.7% in YQ after 3, 6, and 9 days, respectively.

Table 1 exhibits that TPC and AOA increased with the progression of germination time followed by a decrease on the ninth day. The HPLC analysis of phenolic compounds of raw YQ and RQ seeds, and sprouts (grown at 17 ± 1 °C and 90–93% RH) was carried out after 3 and 6 days (Table 2). Among 11 (RQ) and 8 (YQ) quantified compounds, ferulic acid and quercetin were predominant phenolic acid and flavonoids in both quinoa types. On the third day, the number of identified and quantified phenolics in RQ increased to 15 compounds with a valuable increase in some phenolic acids and flavonoids contents of both quinoa types. The highest increase in the polyphenols of RQ and YQ was recorded on the sixth day. The content of phenolic acids and their derivatives, such as gallic, protocatechuic, *p*-hydroxybenzoic, ferulic, *p*-coumaric, rosmarinic, cinnamic acids, and chrysin in RQ and protocatechuic, *p*-hydroxybenzoic, ferulic, and sinapic acids in YQ was increased. Flavonoids, such as rutin, quercetin, and kaempferol gradually increased in RQ and YQ with the progression of the germination period. Basic phytochemicals assays and the HPLC analysis of polyphenols confirmed that the biological activity of RQ and YQ sprouts was suppressed after 6 days of germination. The occurrence of the diseases might be a reason behind the suppressed activity. During the second phase of the research, RQ and YQ sprout extracts were biochemically investigated after 6 days of germination against CCl_4_-induced oxidative stress in an animal model.

Table 3 illustrates the mean values of the relative weight (RW) of livers and kidneys isolated from experimental rats of different treatments after 4 weeks. The liver and kidney RWs of the control rats were 2.83 g and 0.68 g, respectively. CCl_4_ injection significantly increased the RW of the liver. The rat treatment with RQS, YQS, and Si-Vit E reduced inflation and resulted in a non-significant increase in CCl_4_-rats as compared to the NR group. The kidney RW was not dramatically affected by CCl_4_ injection and a non-significant increase was observed in the CCl_4_-R group. The rat treatment with 30 mg GAE kg^−1^ of both RQS and YQS improved liver RW similar to the rats injected with reference drug 30 mg Si-Vit E kg^−1^, as compared to the NR group.

Table 4 describes the hypoglycemic effect of RQS and YQS on FBG of CCl_4_-rats. CCl_4_ injection significantly increased the FBG in rats—that is one of the oxidative stress complications. FBG was reduced in the rats treated with 30 mg kg^−1^ RQS as compared to the rats treated with a similar dose of YQS or Si-Vit E. Liver functions (ALT, AST, and TBILI) of the rats with CCl_4_-induced oxidative stress are also illustrated in Table 4. CCl_4_ significantly increased ALT, AST, and TBILI levels in CCl_4_-R when compared to the NR group. Interestingly, a significant improvement in ALT, AST, and TBILI levels was observed in the rats treated with RQS and YQS, as compared to the NR group. The administration of both RQS and YQS significantly improved the ALT and AST levels compared to CCl_4_-R. RQS efficiently improved the liver functions, as compared to YQS. The Si-Vit E injection significantly attenuated the liver function of rats with CCl_4_-induced oxidative stress. The treatment of 30 mg kg^−1^ of Si-Vit E was found to be better than the same dose of RQS and YQS in recovering ALT, AST, and TBILI levels, but it could not efficiently recover FBG level compared with the NR group.

Table 5 illustrates the effect of RQS and YQS on the blood lipid profile of rats with CCl_4_-induced oxidative stress after 4 weeks. A significant increase in TG, TCh, LDL, and VLDL levels of CCl_4_-R was noted. However, a significant decrease in HDL level was recorded after CCl_4_ injection compared with the NR group. The rat treatment with RQS, YQS, and Si-Vit E significantly affected the TG, TCh, and VLDL levels. RQS, YQS, and Si-Vit E treatments significantly increased the HDL and decreased LDL levels. HDL increase was recorded as 14.52%, 29.21%, and 20.39%, whereas LDL decrease was noted as 60.04%, 68.60%, and 59.06% after Si-Vit E, YQS, and RQS treatments, respectively.

Table 6 depicts that the antioxidant markers (GSH and SOD) were significantly decreased in the CCl_4_-R group, whereas the MDA marker of free radical-mediated lipid peroxidation injury was significantly increased as compared to the NR group. The effects of RQS and YQS antioxidant activity were comparable to the Si-Vit E group. The treatment of rats with RQS and YQS distinctly attenuated the oxidative stress induced by CCl_4_ by reducing MDA and increasing GSH and SOD levels. YQS and RQS reduced MDA percentage by 30.0% and 35.5%, respectively. The percentage of GSH remained as 28.7% and 19.1% whereas SOD percentage was noted as 34.5% and 13.7% for RQS and YQS, respectively. The Si-Vit E treatment more effectively reduced the MDA level of rats with CCl_4_-induced oxidative stress, compared to RQS and YQS

## 4. Discussion

Recently, quinoa seeds have gained considerable attention due to their high nutritional value compared to normal grains. Quinoa seeds have potential health benefits and are abundant in natural antioxidants and phytochemicals [6,8]. Sprouting makes quinoa seeds a super nutritious and flavorful food. Interestingly, sprouted quinoa contains all essential amino acids, fiber, iron, antioxidants, and low inhibitors of phytate and enzymes [28]. The bioactivity of the sprouts is dependent on the duration of their growth, and the peak values were reached on the fourth and sixth days for amaranth and quinoa [11].

In the current study, TPC content in RQS and YQS increased with the progression of germination up to 6 days—that is slightly higher than previously reported [29]. The developmental rate of TPC in RQ was insignificantly higher than YQ—that is similar to Brend et al. [30]. Pasko et al. [11] confirmed a greater increase in the TPC in quinoa sprouts as compared to the seeds of amaranth, indicating that the synthesis of phenolic antioxidants might occur during the germination process in a seed-type dependent manner. Comparing quinoa to amaranth, buckwheat, and wheat, the TPC and AOA were highest in buckwheat and decreased in the order buckwheat > quinoa > amaranth. HPLC–DAD showed that quinoa and buckwheat represent the best sources of polyphenols among the studied seeds. In particular, kaempferol and quercetin glycosides in quinoa seeds increased significantly more than other seeds upon sprouting [31]. Accordingly, the AOA of RQS and YQS were increased in a linear relationship with TPC during the progression of germination time. The correlation between high AOA content in RQS and high TPC has been reported in previous studies [6,8,31]. AOA of RQS and YQS significantly increased up to 6 days and then decreased after 9 days. Similarly, Alvarez-Jubete et al. [31] has also reported a 12.6% decrease in the AOA after a span of 82 h. The increase in the AOA of sprouts is one of the metabolic changes that mainly occurs due to an increase in the activity of self-decomposing enzymes. Other common metabolic changes include improved protein and starch digestion, increased sugar and vitamin B content, and lower levels of protease inhibitors [32]. On the other hand, non-phenolic compounds are among the most potent contributors to AOA in quinoa [33]. Quinoa also contains ascorbic acid, phytic acid, and antioxidant peptides that exhibit antioxidant activity. The decomposition of phytochemicals, such as tannins, ascorbic acid, phytic acid, and antioxidant peptides might be the main reason behind the reduction in TPC and AOA after 6 days of germination [34].

Compared to the previous literature on quinoa, the current data on TC content in YQ were higher than those of RQ. Nevertheless, data of both types were consistent with the results of Ng et al. [35] but higher than the values observed by Tang et al. [36]. The TC content in quinoa and sprouts is much higher than other grains. The presence of lutein and zeaxanthin as the main carotenoids in quinoa can provide greater nutritional and health benefits than other grains [37]. We also noted that TC content in RQS was higher than YQS, which may explain the increase in TPC and AOA. The formation of carotenoids in sprouts during metabolic processes increased the TC content. This increase is due to protein degradation and starch singularity, which led to better extraction with the continuation of the germination cycle. A significant difference was observed (*p* < 0.05) between the total mean of TC in RQ sprouts and YQ sprouts regardless of the germination time [30]. Mastebroek et al. [38] classified quinoa based on the saponin content. Similarly, the current TS data categorized both quinoa types as bitter genotypes containing 4.7 to 11.3 g kg^−1^ DW. These results of TS content are in line with Gomez-Caravaca et al. [39] and Yao et al. [40]. The germination process significantly reduced the TS content in RQS and YQS with the increasing germination period. Development and water hydration caused this reduction, as water penetrates seeds to release saponins through simple diffusion [41]. However, soaking, washing, thermal extrusion, germination, and roasting effectively reduced the bitter taste of saponins [42]. The present data of TA content in RQ were higher than previously reported by Paśko et al. [11]. The TA content is influenced by light/dark cycle, and germination period. Longer germination periods significantly reduce TA, as anthocyanin tincture dissolves in water during seed hydration [11].

Contrarily, the TF contents in both quinoa types were lower in this study than previously reported [8,29]. Paśko et al. [43] has reported exceptionally high TF values (14.82 to 144.3 mg QE 100 g^−1^) as compared to other studies. Sharma et al. [44] and Hirose et al. [45] have, respectively, reported TF values in RQ as 7.05 mg 100 g^−1^ and 3.3–113.3 mg 100 g^−1^. The germination process significantly increased the TF (2–3 times) and TFL (4.4 times) contents, as has also been previously reported [6,8]. Both quinoa types exhibited similar TFL to Hirose et al. [45] but less than that reported by Abderrahim et al. [46]. During the germination process, a significant increase in the TFL content of YQ was observed as compared to RQ. Since quinoa contains abundant TFL compared to other grains; therefore, its consumption is beneficial. It could be concluded that polyphenol content and antioxidant activity in the studied pseudo-cereal seeds during germination support previous studies where polyphenol content and their AOA in seeds increased with the germination period in a linear correlation between TPC content and AOA [11,31,47]. The increase in polyphenols might be due to the activity of self-esterase enzymes that are released during germination and trigger the release of free phenolic compounds located in the seed matrix or linked to the cell wall components [48]. In addition, the biochemical reactions of germs can lead to the synthesis of new phenol compounds [9]. However, certain factors, such as solvents, extraction methods, genotype, soil, environmental conditions, level of maturity at harvest, post-harvest storage conditions, and sprouting conditions also contribute towards variation in polyphenols [10,43,47].

Phenolic compounds consist of phenolic acids, tannins, and flavonoids, which are typically formed in plants, and their quantity may vary with species, conditions, and stage of growth [48]. However, it is difficult to predict the behavior or content of phenolic compounds during the germination cycle as they are a complex biological process [49]. Therefore, to avoid possible polyphenol decomposition, an analysis was immediately performed after extraction without hydrolysis. It facilitated to separately determine aglycons and glycosides as their bioavailability is highly variable. HPLC analysis revealed that ferulic and *p*-hydroxybenzoic acids were more abundant phenolic acids, whereas quercetin, rutin, and kaempferol were more abundant flavonoids in RQ and YQ. Similar results have also been reported by Carciochi et al. [6] and Alvarez-Jubete et al. [31]. The ferulic acid content in RQ and YQ seeds observed in the present study was lower than that found by Repo-Carrasco-Valencia et al. [50] and Miranda et al. [51], and higher than the result of Carciochi et al. [8]. On the third day, gallic acids, apigenin-7-glucoside, apigenin, and scopoletin were detected in RQS, which increased the detectable phenolics up to 15 compounds. Moreover, cateachin was detected in RQS on the sixth day. In total, 16 and 8 polyphenols were detected in RQS and YQS after 6 days of germination, respectively. These results confirmed that the germination process effectively increased the phenolic content in quinoa sprouts as kaempferol and quercetin glycosides in quinoa seeds increased significantly upon sprouting [11,31]. Sinapic acid decreased in YQS but significantly increased in RQS by 108%. This might be due to the type and variety of quinoa. Gallic acid and Cateachin appeared on the sixth day of germination in RQS as a longer germination period improves the decomposition and the extraction of phenolic acids or biochemical reactions in the germinated seeds can lead to the synthesis of new phenol compounds [52]. HPLC analysis indicated that flavonoids, such as rutin, quercetin, and kaempferol in RQ and YQ were quantifiable. The content of rutin, quercetin, and kaempferol was higher in YQS than RQS and increased with the progression of the germination. These results are similar to Alvarez-Jubete et al. [31], and Carciochi et al. [8]. The activity of self-sustaining enzymes manufactured during germination can also lead to the release of phenolic compounds associated with the cell wall, thus increasing their levels. Similarly, the biochemical reactions in the germs can also lead to the synthesis of new phenol compounds [9]. The differences between the values of various studies might be due to the number and amount of phenolic compounds in seed samples that are strongly influenced by the genotype (type/variety), soil, environmental conditions, level of ripening at harvest, and post-harvest storage conditions [6,8].

In the second phase of this study, RQS and YQS extracts were biochemically investigated in rats against CCl4-induced oxidative stress. Carbon tetrachloride (CCl_4_) injection enlarged rat livers by storing fats inside liver cells. The extracts of RQS and YQS distinctly attenuated liver hypertrophy in treated rats and they again attained a normal weight. Interestingly, it was observed that RQS and YQS significantly reduced inflammation and decreased liver RW. Watanabe and Ayugase, [53] also indicated that the intake of Buckwheat sprouts contributed to lower liver weight in type 2 diabetic rats. In the same context, a significant improvement in RW and reduced inflammation of the two kidneys were observed after consuming RQS and YQS. RQS showed better improvement than YQS as it might possess higher antioxidants (TPC, TC, and TA) than YQS after 6 days of germination. These findings coincide with the results of Mbarki et al. [54], who reported the protective effect of fenugreek seeds against CCl_4_-toxicity in kidneys through polyphenol and flavonoid content, which prevented the production of free radicals.

RQS has been reported to significantly decrease the FBG in experimental rats fed with a high fructose diet as compared to control rats [12]. These findings support the results of our study, which confirms that quinoa extracts possess a hypoglycemic effect. RQS strongly reduced FBG as compared to YQS because it contains more polyphenols and antioxidants [43]. Elevated levels of serum enzymes (ALT, AST) activities indicate cellular leakage and loss of functional integrity of cell membranes in the liver. The administration of RQS and YQS significantly improved the levels of liver enzymes (ALT, AST). These results are consistent with Halaby et al. [55] who indicated the attenuation effect of quinoa seeds on ALT (30%) and AST (40%) enzymes in rats with induced hypercholesterolemia. Similarly, Saxena et al. [56] confirmed the effects of quinoa extracts on elevated serum ALT and AST enzymes in rats against CCl4-induced oxidative stress. The TBILI indicates the liver injury and CCl_4_ had a significantly higher level than NR and treated groups (Si-Vit E, RQS, and YEQ). In this regard, the RQS was found to be more efficient than YQS. Previous studies have also indicated that RQS contains anthocyanins, betacyanins, and flavonoids (quercetin and rutin) in valuable amounts. These compounds have been proven to perform antioxidative and anti-inflammatory effects in rats with hepatic damage [57].

Elevated levels of serum triglycerides and cholesterol in the CCl_4_-R group indicates impaired fat metabolism due to hepatic damage. Groups fed with RQS and YQS extracts significantly differed to the CCl_4_-R group. These results approve of the findings of Farinazzi-Machado et al. [58] who reported that daily intake of quinoa pills for 30 days significantly decreased triglycerides. RQS was found better than YQS in improving TG, TCh, LDL, and VLDL levels. This might be due to the rich contents of phenols, antioxidants, carotenoids, and saponins. Several clinical studies have indicated that quinoa consumption can lower cholesterol due to the presence of main ingredients (proteins, fibers, vitamins), tocopherol and carotenoids, minerals (iron, zinc, and magnesium), saponins, plant sterols, and polyphenols [46,47,59].

ROS increases the risk of tissue damage and causes lipid peroxidation, as determined by the catabolite malondialdehyde marker [35]. Previous studies showed that chronic CCl_4_ i.p. injection significantly reduced SOD, CAT, GPx, and GSH activities, but significantly increased the MDA level [60]. The administration of 30 mg kg^−1^ RQS was more efficient than YQS, which might be due to the high content of polyphenols and AOA in RQS. The enzymatic antioxidant defense system, such as SOD and glutathione enzymes are important scavengers of active radicals [57]. RQS and YQS attenuated GSH, SOD, and MDA levels close to the NR. Previous studies have reported that the consumption of quinoa seeds and sprouts led to increased GSH levels [10,53]. De Carvalho et al. [59] have also shown that the consumption of about 25 g of quinoa chips by postmenopausal women increased GSH levels. The results are consistent with another study where quinoa seeds reduced oxidative stress in plasma, heart, kidneys, liver, spleen, lung, testicle, and pancreas in fructose-fed mice, and lowered MDA in plasma [12]. Due to the presence of more polyphenols, the RQS efficiently reduces complications related to oxidative stress. Therefore, RQS provides better liver protection than YQS by blocking the development of liver fibrosis via suppressing TGF-β1 [57]. Its mechanism was involved in antioxidative system activation, suppressing the TNF-α/IL-6 pathway, and blocking the TGF-β1 pathway [57]. However, valuable identified phenolics and flavonoids in RQ sprouts as bioactive compounds (see Table 2) may have an efficient activity to prevent CCl_4_-induced oxidative stress and liver inflammation, since RQS had a greater effect than YQS extracts.

Remarkably, the used level of sprout extracts is achievable by including germinated quinoa in the diet. Using the data in Table 1 (TPC content in RQ and YQ sprouts), the administration level from rat experiments (30 mg kg^−1^), and the presented equation by Reagan-Shaw et al. [15], a human portion was calculated to be 1.67 g and 1.88 g quinoa sprouts per kg^−1^ of human body weight for RQ and YQ sprouts, respectively. Therefore, the incorporation of quinoa sprouts into the diet to get the potential health activity is highly encouraged.

## 5. Conclusions

Liver injury causes liver cirrhosis and fibrosis. In the current study, CCl_4_ i.p. injection not only enhanced the liver function but also affected the blood profile and substantially disrupted antioxidant systems by lowering the SOD and GSH activity. Quinoa and its sprouts are seen as an innovation and, therefore, its therapeutic and nutritional aspects should be given more attention. The results indicated that polyphenols and AOA increased in RQS and YQS after 6 days of germination. HPLC analysis revealed that RQS contains more bioactive compound diversity than YQS. The findings of the study proved that quinoa sprouts attenuated the adverse effects of induced oxidative stress. Interestingly, the treatment of rats with 30 mg of GAE kg^−1^ significantly reduced FBG, ALT, AST, and TIBIL and improved liver inflammation. Furthermore, RQS and YQS improved the blood profile by significantly decreasing LDL and VLDL and increasing HDL. Moreover, RQS and YQS extracts significantly reduced MDA and efficiently enhanced GSH and SOD activities in oxidative stress-induced rats, as compared to CCl_4_-rats. In conclusion, RQS and YQS provide naturally synthesized polyphenols and possess superior antioxidant activity. Their ethanolic extracts may have promising effects and various health benefits against oxidative stress. Therefore, incorporating quinoa sprouts as functional food ingredients should be considered, and scaling-up its production is beneficial.

## Figures and Tables

**Table 1:** Phytochemical content in yellow and red quinoa seeds and sprouts during 9 days of germination at 17 ± 1 °C and 90–93% relative humidity.

Phytochemicals	Germination Period (Days)							
	RQ				YQ			
	0-d	3-d	6-d	9-d	0-d	3-d	6-d	9-d
Total phenolic content (mg GAE 100 g^−1^)	105.16 ± 9.66 ^d^	257.10 ± 28.81 ^b^	293.35 ± 14.80 ^a^	251.23 ± 13.77 ^c^	112.42 ± 5.65 ^d^	190.43 ± 3.00 ^c^	259.02 ± 5.84 ^a^	221.74 ± 5.92 ^b^
DPPH-RSA (m mol TE 100 g^−1^)	4.35 ± 0.70 ^c^	6.02 ± 0.17 ^b^	7.39 ± 0.14 ^a^	7.20 ± 0.04 ^a^	3.00 ± 0.16 ^c^	3.71 ± 0.51 ^b^	5.26 ± 0.17 ^a^	3.01 ± 0.33 ^c^
Total carotenoids (mg 100^−1^)	2.22 ± 0.07 ^c^	7.27 ± 0.78 ^b^	15.58 ± 0.92 ^a^	17.14 ± 2.67 ^a^	3.44 ± 0.16 ^c^	4.93 ± 0.34 ^b^	8.11 ± 1.24 ^a^	7.48 ± 0.95 ^a^
Total saponins (mg g^−1^)	6.53 ± 0.07 ^a^	6.10 ± 0.78 ^a^	2.66 ± 0.43 ^b^	0.93 ± 0.27 ^c^	6.10 ± 0.43 ^a^	3.52 ± 0.34 ^b^	0.93 ± 0.24 ^c^	0.50 ± 0.15 ^d^
Total anthocyanin (mg 100^−1^)	179.96 ± 15.81^a^	160.75 ± 11.70 ^ab^	125.90 ± 23.49 ^bc^	91.82 ± 4.98 ^c^	-	-	-	-
Total flavonoids (mg QE 100 g^−1^)	7.05 ± 0.09 ^d^	9.59 ± 0.10 ^c^	10.38 ± 0.38 ^b^	13.17 ± 0.27 ^a^	9.04 ± 0.45 ^c^	19.24 ± 0.60 ^b^	24.00 ± 2.10 ^a^	24.36 ± 1.14 ^a^
Total flavonols (mg QE 100 g^−1^)	5.88 ± 0.42 ^d^	7.23 ± 0.45 ^c^	8.06 ± 0.05 ^b^	9.36 ± 0.26 ^a^	3.69 ± 0.25 ^d^	11.06 ± 0.97 ^c^	20.66 ± 0.33 ^b^	22.24 ± 1.06 ^a^

RQ: red quinoa; YQ: yellow quinoa; GAE: Gallic acid Equivalent; RSA: radical scavenging activity; QE: Quercetin Equivalent, ^a, b, c^ and ^d^: The values with the same superscript letter are not significantly different at *p* < 0.05.

**Table 2:** High-performance liquid chromatography-diode array detection (HPLC-DAD) based identification and quantification of phenolic compounds in yellow and red quinoa seeds and sprouts during 6 days of germination at 17 ± 1 °C and 90–93% relative humidity.

Compound		Rt	Phenolics (µg g^−1^)					
			RQ			YQ		
			0-d	3-d	6-d	0-d	3-d	6-d
1	Gallic acid	4.413	-	0.47	-	-	-	-
2	Protocatechuic acid	7.593	-	11.11	9.87	14.29	9.66	39.01
3	*p*-hydroxybenzoic acid	11.000	1.00	39.25	4.80	13.40	10.37	19.71
4	Cateachin	12.604	34.57	-	46.79	-	-	-
5	Scopoletin	25.224	-	46.65	15.85	-	-	-
6	Ferulic acid	26.442	-	17.33	37.19	28.42	55.14	81.15
7	Sinapic acid	28.282	47.13	4.93	57.33	5.94	9.96	12.34
8	*p*-coumaric acid	30.865	18.18	0.98	17.21	-	-	-
9	Rutin	32.58	0.59	1.56	8.69	2.23	2.33	3.85
10	Apigenin-7-glucoside	36.827	1.05	1.18	1.83	-	-	-
11	Rosmarinic acid	38.004	-	1.46	1.28	2.36	3.23	2.30
12	Cinnamic acid	41.33	1.91	2.48	4.07	-	-	-
13	Quercetin	44.435	1.28	1.98	3.13	5.69	2.95	7.16
14	Apigenin	48.156	1.51	0.52	2.56	-	-	-
15	Kaempferol	48.758	-	2.09	0.98	0.41	1.10	2.69
16	Chrysin	55.123	0.43	2.58	1.36	-	-	-

-: not detected by HPLC.

**Table 3:** Effect of quinoa extracts on the relative weight of livers and kidneys of rats with CCl_4_-induced oxidative stress (mean ± SE), *n* = 6.

Groups	Liver Relative Weight (%)	Kidney Relative Weight (%)
NR	2.83 ± 0.18 ^b^	0.68 ± 0.04 ^a^
CCl_4_-R	3.47 ± 0.09 ^a^	0.71 ± 0.05 ^a^
CCl_4_-R + Si-Vit E	2.89 ± 0.12 ^b^	0.69 ± 0.02 ^a^
CCl_4_-R + YQS	2.85 ± 0.08 ^b^	0.63 ± 0.01 ^ab^
CCl_4_-R + RQS	2.76 ± 0.03 ^b^	0.61 ± 0.01 ^b^

NR: received an intraperitoneal injection of fresh olive oil; CCl_4_-R: received an intraperitoneal injection of a freshmixture of equal volumes of CCl_4_ and olive oil; RQS: Red quinoa sprouts; YQS; Yellow quinoa sprouts. ^a^ and ^b^: The values with the same superscript letter are not significantly different at *p* < 0.05.

**Table 4:** Effect of quinoa extracts on FBG, ALT, AST, and TBILI of rats with CCl_4_-induced oxidative stress (mean ± SE), *n* = 6.

Groups	FBG (mg dL^−1^)	ALT (u L^−1^)	AST (u L^−1^)	TBILI (mg dL^−1^)
NR	90.00 ± 1.00 ^b^	72.67 ± 3.14 ^c^	182.33 ± 3.31 ^d^	0.22 ± 0.05 ^c^
CCl_4_-R	108.60 ± 1.46 ^a^	104.00 ± 2.27 ^a^	259.33 ± 12.42 ^a^	0.85 ± 0.11 ^a^
CCl_4_-R + Si-Vit E	107.87 ± 1.15 ^a^	71.23 ± 2.37 ^c^	184.77 ± 1.29 ^d^	0.46 ± 0.13 ^b^
CCl_4_-R + YQS	106.17 ± 0.79 ^a^	97.32 ± 3.15 ^b^	199.00 ± 1.04 ^b^	0.55 ± 0.09 ^b^
CCl_4_-R + RQS	87.76 ± 1.92 ^bc^	97.33 ± 4.55 ^b^	188.33 ± 1.29 ^c^	0.49 ± 0.08 ^b^

FBG: Fasting blood glucose; ALT: Alanine aminotransferase; AST: Aspartate aminotransferase; TBILI: Total bilirubin. ^a, b, c^ and ^d^: The values with the same superscript letter are not significantly different at *p* < 0.05.

**Table 5:** Effects of quinoa extracts on blood lipids profile of rats with CCl_4_-induced oxidative stress (mean ± SE), *n* = 6.

Groups	TG (mg dL^−1^)	TCh (mg dL^−1^)	HDL (mg dL^−1^)	LDL (mg dL^−1^)	VLDL (mg dL^−1^)
NR	33.67 ± 3.09 ^b^	50.33 ± 2.51 ^c^	37.23 ± 1.69 ^b^	6.37 ± 0.93 ^b^	6.73 ± 0.62 ^b^
CCl_4_-R	37.67 ± 2.22 ^a^	56.76 ± 0.52 ^a^	34.03 ± 0.38 ^c^	15.19 ± 0.26 ^a^	7.53 ± 0.44 ^a^
CCl_4_-R + Si-Vit E	34.12 ± 0.76 ^b^	51.64 ± 1.11 ^b^	38.97 ± 0.86 ^b^	6.07 ± 0.16 ^b^	6.60 ± 0.15 ^b^
CCl_4_-R + YQ	33.00 ± 0.77 ^bc^	55.33 ± 1.12 ^a^	43.97 ± 0.87 ^a^	4.77 ± 0.16 ^c^	6.60 ± 0.16 ^b^
CCl_4_-R + RQ	30.00 ± 2.51 ^c^	51.67 ± 2.05 ^b^	40.97 ± 1.68 ^b^	4.70 ± 0.52 ^c^	6.00 ± 0.50 ^b^

TG: Triglycerides, TCh: Total cholesterol, HDL: High-density lipoprotein, LDL: Low-density lipoprotein, VLDL: Very low-density lipoprotein, ^a, b^ and ^c^: The values with the same superscript letter are not significantly different at *p* < 0.05.

**Table 6:** Effects of quinoa extracts on oxidative stress biomarkers in rats with CCl_4_-induced oxidative stress (mean ± SE), *n* = 6.

Groups	GSH [µmol L^−1^]	SOD [u L^−1^]	MDA [n mol mL^−1^]
NR	117.66 ± 2.03 ^a^	85.53 ± 0.42 ^a^	9.12 ± 0.18 ^d^
CCl_4_-R	85.10 ± 2.77 ^e^	60.60 ± 0.80 ^e^	16.53 ± 0.13 ^a^
CCl_4_-R + Si-Vit E	106.16 ± 1.22 ^c^	79.11 ± 0.73 ^c^	8.79 ± 0.05 ^e^
CCl_4_-R + YQ	101.38 ± 0.77 ^d^	68.28 ± 1.33 ^d^	10.67 ± 0.08 ^c^
CCl_4_-R + RQ	109.52 ± 1.49 ^b^	81.50 ± 0.33 ^b^	11.57 ± 0.11 ^b^

GSH: glutathione; SOD: Superoxide dismutase; MDA: Malonaldehyde. ^a, b, c, d^ and ^e^: The values with the same superscript letter are not significantly different at *p* < 0.05.

## Abbreviations

ALT: Alanine aminotransferase; AOA; Antioxidant activity; AST; Aspartate aminotransferase; CAT; Catalase; FBG; Fasting blood glucose; GPx; Glutathione peroxidase; HDL; High-density lipoproteins; LDL; Low-density lipoproteins; MDA; Malonaldehyde; ROS; Reactive oxygen species; RQ; Red quinoa; RQS; Red quinoa sprouts; SOD; Superoxide dismutase; TA; Total anthocyanins; TBA; Thiobarbituric acid; TBILI; Total bilirubin; TC; Total carotenoids; TCh Total cholesterol; TF; Total flavonoids; TFL; Total flavonols; TG; Triglycerides; TPC; Total phenolic compounds; TS; Total saponins; VLDL; Very Low-density lipoproteins; YQ; Yellow quinoa; YQS; Yellow quinoa sprouts.
